# Adolescent-and-Young-Adult-Onset Multisystem Langerhans Cell Histiocytosis With Central Nervous System Involvement: A Case Report

**DOI:** 10.7759/cureus.91053

**Published:** 2025-08-26

**Authors:** Sruthi Dontu, Mei Zheng, Michael Chahin

**Affiliations:** 1 Department of Internal Medicine, Medical College of Georgia at Augusta University, Augusta, USA; 2 Department of Pathology, Medical College of Georgia at Augusta University, Augusta, USA; 3 Department of Hematology/Oncology, Summit Cancer Care, Savannah, USA

**Keywords:** adolescent-and-young-adult, central nervous system, cladribine treatment, langerhans cell histiocytosis, multisystem, skeletal system

## Abstract

Langerhans cell histiocytosis (LCH) is a proliferative disorder causing normally immune-responsive Langerhans cells to abnormally accumulate in various tissues and organs. Most available data on LCH is derived from pediatric populations, with limited literature focusing on adult LCH, which is rarer. Multisystem involvement in LCH, including central nervous system (CNS) involvement, is often higher risk and poorer prognosis. Standardized treatment recommendations remain limited, particularly in adolescent and young adult (AYA) populations. Discussed below is a case of AYA-onset multisystem LCH with CNS and skeletal system involvement, which was successfully treated with cladribine therapy. An 18-year-old male with no significant past medical history presented with left orbital pain and swelling. Laboratory, imaging, and biopsy results were consistent with a diagnosis of multisystem LCH. The patient was started on cladribine at 0.1 mg/kg/day for 7 days, along with Pneumocystis pneumonia prophylaxis and symptomatic management of facial pain and headaches. After four cycles of cladribine therapy, the patient exhibited symptomatic resolution and complete response of CNS and skeletal lesions. This highlights one potential therapeutic approach yielding a favorable outcome in a borderline case of AYA-onset LCH without targetable mutations on tissue next-generation sequencing (NGS). It also underscores the need for systemic therapy in patients with CNS involvement to avoid future long-term neurodegenerative complications. Further prospective studies and clinical trials are warranted to yield standardized treatment regimens for adult patients with LCH, particularly with multisystem involvement, including CNS.

## Introduction

Langerhans cell histiocytosis (LCH) is a rare proliferative disorder that involves abnormal clonal expansion of CD1a and CD207-positive myeloid-committed hematopoietic precursors, which further differentiate into monocytes and finally circulate as Langerhans cells. Langerhans cells are specific types of dendritic cells that normally reside in the skin and are involved in the body’s immune response to help fight infections. However, specific mutations can alter the normal function of these cells, causing them to abnormally accumulate in various tissues, including but not limited to the skin, liver, spleen, lung, gastrointestinal (GI) tract, central nervous system (CNS), and skeletal system. The pathogenic cells are defined by constitutive activation of the MAP kinase signaling pathway through mutations in genes like BRAF, MEK1, and MAP2K1 [1].

Most LCH data are derived from studies in children, with limited literature examining adult LCH. The median age at diagnosis of LCH in children is three years, and the reported incidence ranges from 2.6 to 8.9 cases per million children younger than 15 years per year [2]. It is even rarer in adults, with the exact incidence undefined but estimated at approximately 1-1.5 cases per million annually [3]. Other histiocytic disorders in adults include Erdheim-Chester Disease (ECD), with an estimated incidence of 3.5 cases per 10 million adults annually, and Malignant histiocytosis (MH) with an estimated incidence of less than one case per million annually [4,5]. Regarding median age at diagnosis of adult LCH, one retrospective study estimated this at 43 years [6]. A recent classification system proposed in the setting of increasing evidence of LCH in adults categorized LCH into four main categories: unifocal (solitary lesion involving any organ), single-system pulmonary (isolated lung involvement, predominantly smoking related), single-system multifocal (>1 lesion involving any organ), and multisystem (>=2 organ/system involvement) [3]. In the retrospective study reported above, around 38% of patients exhibited multisystem involvement [6].

While patients without organ dysfunction have excellent survival rates, those with organ dysfunction can have mortality rates up to 20% [1]. The prevalence of CNS involvement in LCH, specifically, has been found to range from 3.4% to 57% and prognosis is typically poor [7]. With multisystem disease, 60% of patients have a chronic course, 30% achieve remission, and mortality is up to 10%. Standardized treatment is yet to be established, and treatment failure is also associated with increased mortality and morbidity [3]. Additionally, there is a paucity of data and treatment recommendations on the onset of LCH in adolescent and young adult (AYA) populations. We report a case of AYA-onset multisystem LCH with CNS and skeletal system involvement, which was successfully treated with cladribine therapy as per suggested consensus recommendations.

## Case presentation

An 18-year-old male with no significant past medical history presented with left orbit pain, lateral facial pain, and left periorbital swelling. Vital signs were stable. Physical exam revealed left-sided facial swelling, left facial pain worst in the V1 distribution, and no gross focal neurological deficits. Laboratory results revealed elevated erythrocyte sedimentation rate (ESR) and C-reactive protein (CRP) at 22 mm/hour and 0.624 mg/dL, respectively. No other laboratory abnormalities were noted. 

**Figure 1:**
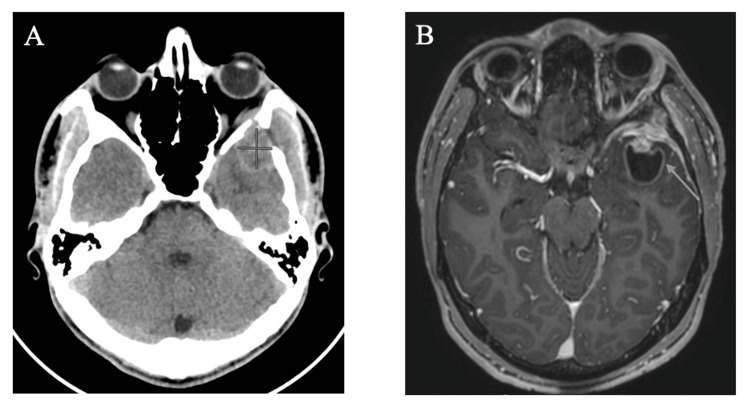
Initial CT head and MRI brain findings. (A) CT head shows an intracranial mass centered in the left greater sphenoid area; (B) MRI brain shows the same mass measuring 2.9 cm x 2.3 cm x 2.6 cm, extending into the surrounding area. CT, computed tomography; MRI, magnetic resonance imaging

Computed tomography (CT) head revealed an intracranial mass, and subsequent magnetic resonance imaging (MRI) brain revealed an avidly enhancing and mildly diffusion restricting mass lesion centered in the left greater sphenoid involving the left middle cranial fossa, infratemporal fossa, masticator space, and orbit, measuring 2.9 cm x 2.3 cm x 2.6 cm, concerning for aggressive neoplasm. The mass also exhibited epidural extension of enhancement in the left middle cranial fossa with likely intraparenchymal invasion of the left temporal pole with peritumoral cyst, and involvement of the overlying temporalis muscle, lateral left orbit, and left infratemporal fossa (Figure 1). CT chest/abdomen/pelvis did not show evidence of metastatic disease. Lumbar puncture (LP) cerebrospinal fluid (CSF) flow cytometry, to exclude CSF/leptomeningeal involvement and CNS lymphoma in the setting of headache and mass effect, was negative, and fluid studies were unremarkable. Neurosurgery evaluated the patient and recommended no acute surgical intervention. For symptomatic control, the patient received oral dexamethasone 4 mg twice daily for one week, followed by 4 mg daily for one week, and oral gabapentin 100 mg thrice daily for facial pain and headaches. He was also started on oral levetiracetam 1 g daily for seizure prophylaxis. 

**Figure 2:**
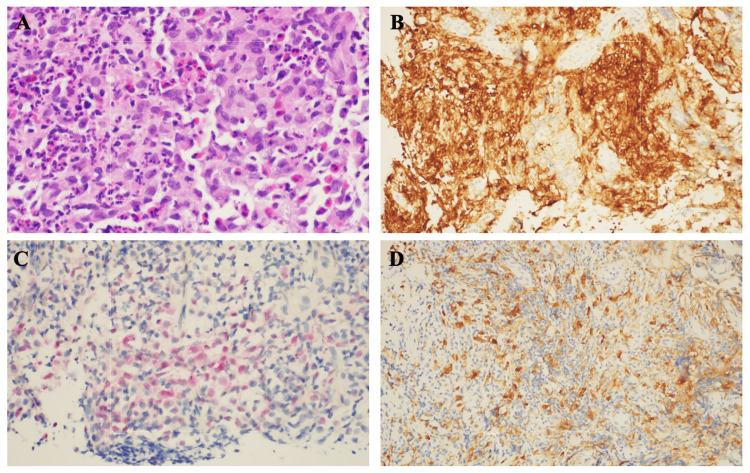
Left sphenoid mass pathology and special stain results. (A) The specimen shows clusters of large, round to oval histiocytes with grooved to convoluted nuclei (H&E stain, 40X objective lens) in a background of eosinophils, histiocytes, and small lymphocytes. (B) The lesional cells show a Langerhans cell phenotype (immunostain) CD1a+ (membranous, 20X objective lens). (C) S100 stain (nuclear and cytoplasmic, 20X objective lens). (D) CD163 stains scattered histiocytes (20X objective lens).

**Figure 3:**
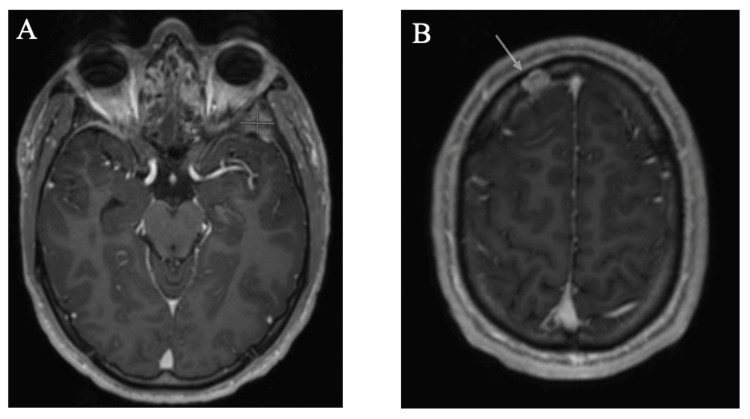
MRI brain findings after three cycles of cladribine. (A) Left sphenoid mass size reduction and resolution of mass effect. (B) New right frontal calvarium lesion (arrow) without intracranial extension. MRI, magnetic resonance imaging

**Figure 4:**
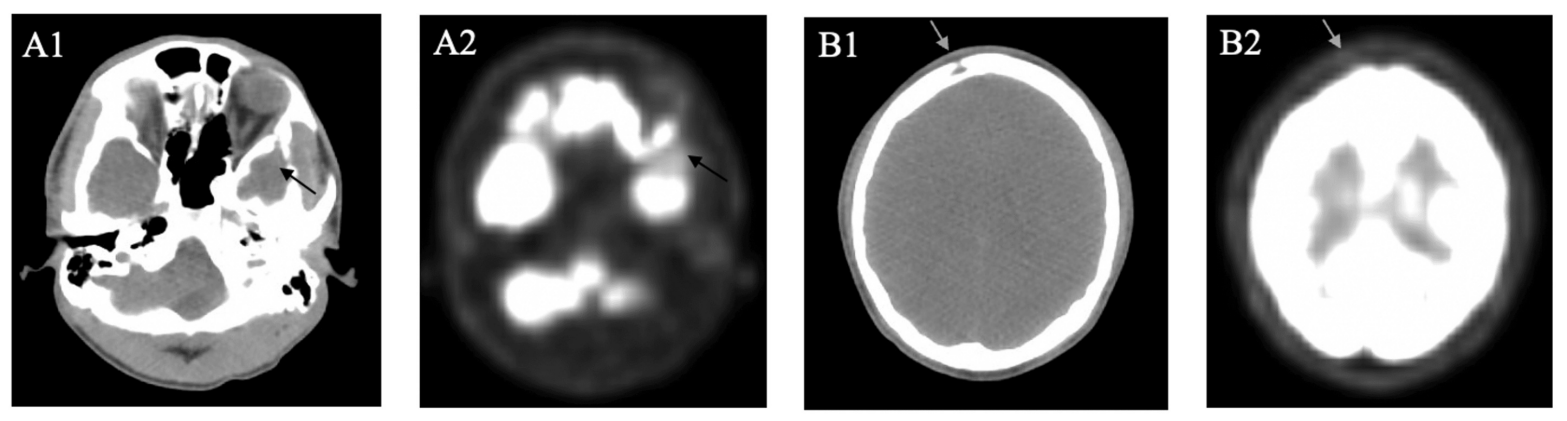
PET/CT findings after three cycles of cladribine. (A1) CT, (A2) PET: Left sphenoid mass size reduction (arrow). (B1) CT, (B2) PET: Interval size increase of the right frontal lytic lesion (arrow). PET/CT, positron emission tomography/computed tomography

Biopsy of the mass revealed LCH (Figure 2), and genetic tissue testing for NRAS, BRAF, and EGFR returned negative. Patient was started on cladribine (2-CdA) 0.1 mg/kg/day x 7 days, along with Pneumocystis pneumonia prophylaxis with trimethoprim/sulfamethoxazole (TMP/SMX) 800 mg-160 mg, given his high risk of infection. He completed a total of four cycles. After three cycles of induction, brain MRI showed interval development of a new enhancing lesion in the right frontal calvarium without intracranial extension, a significant interval reduction in the size of the left sphenoid lesion, and interval resolution of the mass effect on the left temporal lobe (Figure 3). At this point, the patient’s headaches and facial swelling resolved, and steroids were discontinued. A later positron emission tomography (PET)/CT showed an interval increase in the size of the lytic lesion in the right frontal bone, but a decrease in the metabolic activity of the left middle skull base soft tissue lesion (Figure 4). Following this, the patient declined radiation for the frontal lobe lesion. 

**Figure 5:**
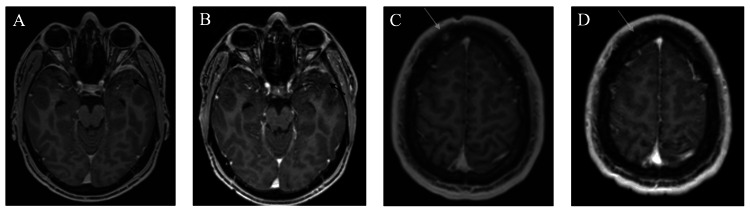
MRI brain findings after four cycles of cladribine. (A) Interval decrease in size of left sphenoid mass, followed by (B) stable size; (C) interval decrease in size of the right frontal calvarium lesion (arrow), followed by (D) stable size (arrow). MRI, magnetic resonance imaging

**Figure 6:**
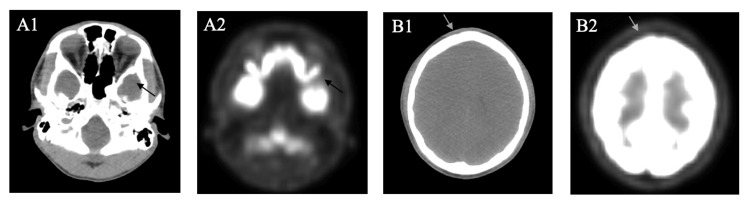
PET/CT findings after four cycles of cladribine. (A1) CT, (A2) PET: Stable size of the left sphenoid lesion (arrow) with near-resolution of abnormal FDG activity; (B1) CT, (B2) PET: Stable size of the right frontal bone lesion (arrow) with decrease in FDG activity. PET/CT, positron emission tomography/computed tomography; FDG, fluorodeoxyglucose

After four cycles of induction, PET/CT and MRI scans showed a complete metabolic response with stable findings in the primary left sphenoid region, along with a stable size of the right frontal lobe lesion with some reduction in PET avidity (Figures 5-6). At this point, the patient was completely asymptomatic and tapered off levetiracetam, TMP/SMX, and gabapentin. Repeat imaging in six months showed a complete response. The patient currently remains asymptomatic with no further progression of CNS lesions. He is being monitored with PET/CT and MRI brain scans every three to six months.

## Discussion

LCH is primarily a pediatric disorder, usually occurring in younger children and commonly involving soft tissues such as the skin and bone. Treatment of multisystem pediatric LCH often involves systemic chemotherapy, while the discovery of BRAF mutations has heralded advances in treatment with targeted agents [3]. These advances in risk-adapted treatment have had an overall improvement in outcomes, particularly in cases of disseminated LCH, although these improvements appear to have favored children over adults, with five-year relative survival rates of 90% versus 70%, respectively [8].

Within the adult LCH population, CNS involvement can be slightly more common with the concomitant presence of BRAF V600E mutations [3,9]. However, orbital involvement, as was found in this patient, is rare [10]. Given the patient’s CNS symptoms of headache and compression of mass on brain parenchyma, CSF/leptomeningeal involvement and CNS lymphoma were considered but ruled out with negative LP CSF flow cytometry and fluid studies. A recent retrospective series on adult LCH discovered BRAF mutations in 58% of the patients [11]. Another study showed that those with the BRAF mutation were more likely to have CNS involvement than those without the mutation and were associated with lower overall survival (OS) rates [6,9]. Recent advances are defining the emerging role of targeted agents in LCH in adults as well. However, there is a dearth of adult LCH prospective data, thus limiting the development of optimal therapeutic strategies for this population, particularly in the case of CNS disease, which is considered high-risk with poorer prognosis. Additionally, pediatric regimens may cause greater toxicities in adult patients who may have poorer baseline functional statuses and greater comorbidities, and risk-based stratification systems used in pediatric populations have also not been validated in adults [3]. The largest trial evaluated a two-drug combination regimen of methotrexate and cytarabine with a response rate of 88% and a three-year progression rate of 32%. However, around 50% of those patients developed febrile neutropenia and 33% developed high-grade thrombocytopenia [12].

Recent consensus recommendations suggest that for adults with CNS disease, cladribine, higher doses of cytarabine, IV methotrexate-based regimens, or kinase inhibitors are preferred [3]. For adults with neurodegenerative disease, BRAF/MEK-inhibitor- or cytarabine-based chemotherapy may be preferred based on limited pediatric experience, but the literature is lacking [13]. A 1999 prospective trial using cladribine in adults with LCH showed a response rate of 75% with a median duration of three years and a febrile neutropenia rate of 15% [14]. Recent studies have reiterated the favorable response rates among patients with LCH who were treated with cladribine [15,16]. A phase II clinical trial is currently underway in adult patients with LCH to investigate clofarabine, a drug that has been effective in pediatric LCH, which may be a promising future therapeutic agent in adult patients with LCH [17].

Given that the median age of LCH onset is three years in children and around 40 years in adults, and that treatment recommendations differ between adults and pediatric patients due to differing presentations and limited data in adult populations, this case of LCH onset at 18 years of age was atypical. This complex presentation made it difficult to decide which treatment would be optimal to confer a high response rate. Given that the disease had progressed beyond involving two systems (CNS and skeletal), specific local therapies, including surgical resection, were deferred. With regards to long-term CNS complications, LCH-associated abnormal CNS symptoms (LACS) is a reported neurodegenerative syndrome with variable severity that occurs at a higher frequency in patients with multisystem CNS disease, diabetes insipidus, and lesions involving the skull-base and orbit, the latter of which applies to our patient [1]. As a result, skull-based lesions, in particular, have been an indication for systemic therapy such as cladribine, rather than localized resection. Further, although the patient was technically in the adult subgroup, he tested negative for targetable mutations and thus BRAF inhibitors were deferred. Ultimately, given the presence of multisystem CNS disease and lack of targetable mutations, patient was started on cladribine therapy. He was treated with four cycles and exhibited a complete response with symptomatic resolution. This provides support to recent studies that have shown that limiting cladribine treatment to four cycles may reduce the rate of severe cytopenias, and that maximum response is usually attained within four cycles of treatment [15]. The patient is also undergoing PET/CT and brain MRI q3-6 months for long-term monitoring, in accordance with current recommendations for LCH patients with multisystem disease [3].

## Conclusions

This case demonstrates one potential therapeutic approach - four cycles of cladribine - yielding a favorable outcome in a case of AYA-onset LCH that is borderline between childhood and adulthood age. It underscores the need for systemic chemotherapy in patients with multisystem disease and CNS involvement to prevent long-term complications of disease including neurodegenerative syndromes, focal deficits, and possible irreversible damage to important brain structures. This is essential for a favorable long-term prognosis which is often difficult to obtain in adult multisystem LCH.

The case also highlights the role of cladribine therapy in adults or adolescents without targetable BRAF mutations on tissue NGS who may not benefit from therapies like BRAF inhibitors. In these patients, cladribine can potentially offer symptomatic resolution in addition to CNS and skeletal lesion response. This regimen offers a viable option for similarly presenting patients at the cusp between childhood and adulthood where treatment recommendations are limited. Further prospective studies and clinical trials are warranted to yield standardized treatment regimens for adult patients with LCH and particularly with multisystem involvement including CNS.
